# Nuclear Migration: An Indicator of Plant Salinity Tolerance *in vitro*

**DOI:** 10.3389/fpls.2019.00783

**Published:** 2019-06-12

**Authors:** Adel M. Elmaghrabi, Dennis Francis, Hilary J. Rogers, Sergio J. Ochatt

**Affiliations:** ^1^School of Biosciences, Cardiff University, Cardiff, United Kingdom; ^2^Agroécologie, AgroSup Dijon, INRA, Université Bourgogne Franche-Comté, Dijon, France

**Keywords:** abiotic stress, cell morphometry, cell suspensions, *Medicago truncatula*, *Nicotiana tabacum*, nucleus position, salinity tolerance

## Abstract

In order to understand the mechanisms underlying acquisition of tolerance to salinity, we recently produced callus tissues of tobacco and *Medicago truncatula* resistant to NaCl-induced salt stress following application of a step-up recurrent selection method. The effects of salinity on cell size are known, but those on cell morphometry including cell and nuclear surface area and position of nuclei within salt stress resistant cells were never studied before. This work fills that gap, using suspension cultured cells of *M. truncatula* A17 initiated from callus, and *Nicotiana tabacum* BY-2 cell line resistant to increasing NaCl concentrations up to 150 mM NaCl. The surface area of salinity resistant cells of *M. truncatula* A17 and *N. tabacum* BY2 and their nuclei, produced by step-up recurrent selection, were reduced, and cells elongated as NaCl increased, but these parameters proved to be unreliable in explaining cell survival and growth at high NaCl. Conversely, nuclei of resistant cells migrated from the center to the periphery of the cytoplasm close to the walls. Nuclear marginalization was for the first time observed as a result of salt stress in plant cells, and could be a novel helpful morphological marker of acquisition of salinity tolerance.

## Introduction

Increased soil salinity is a world-wide problem, hence there is a need to develop more salinity-resistant crop cultivars (Ochatt, 2015). The mechanisms of plant salt tolerance *in vivo* have been investigated at the molecular, cellular, and whole plant levels (Munns and Tester, 2008). *In vitro* selection for salt tolerance has focused on cellular (Davenport et al., 2003) and genetic (Elmaghrabi et al., 2013) mechanisms involved in salt tolerance using selected NaCl-tolerant cell lines, while gene transfer has also been successfully exploited very recently to generate salt (Confalonieri et al., 2019) and water stress (Confalonieri et al., 2014; Alcântara et al., 2015; Duque et al., 2016) tolerance in *M. truncatula*. Alternative methods to exploit *in vitro* stress to characterize the biology and genetic diversity of early stage seedling growth (Parida and Das, 2005; Elmaghrabi et al., 2013), as well as effects of salinity stress on plant morphology, have also been extensively studied (Parida and Das, 2005; Claeys et al., 2014; Golkar et al., 2017; Negrão et al., 2017). However, only effects of salinity on cell size have been examined to date (Kurth et al., 1986) while those on cell morphometry have not.

Nuclear positioning is important during cell division, mediated by the three cytoskeletal filament systems, F-actin, intermediate filaments (IF), and microtubules (Ingber, 2003). In a recent review, Gundersen and Worman (2013) examined the sparse knowledge and understanding of the reasons and effects of cell movement and of the position of their nuclei within the cytoplasm. Given that nuclear positioning has been reported to reflect an interference with the proteins involved in nuclear movement (Maniotis et al., 1997; Folker et al., 2011), they hypothesized that this may then inhibit a number of cellular activities. These include an effect on the organization and mechanical properties of the cytoplasm with a concomitant impact on cytoplasmic signaling and on the accessibility of the nucleus to the associated signaling pathways (Dahl et al., 2004; Gundersen and Worman, 2013). However, to our knowledge, this hypothesis that nuclear movement may regulate cellular signaling pathways and responses to stress (Gundersen and Worman, 2013) has never been directly tested to date, be it with animal or plant cells.

Recently we developed a step-up selection method in *M. truncatula*, for obtaining embryogenic calli under increasing salt stress. Within 5 months, different developmental patterns of callus varying between embryogenic to a non-regenerative condition were observed, correlated with a differential nuclear DNA content and biochemical profile (Elmaghrabi and Ochatt, 2006; Elmaghrabi et al., 2013). Callus growth was significantly impaired at ≥100 mM NaCl but green callus was observed up to 100–150 mM NaCl, coincident with healthy growth despite the high salinity. However, 250 and 350 mM NaCl were lethal to most cells, and only small clusters of cells survived. To assess how this step-up approach affected cellular morphology, it was adapted and applied to *M. truncatula* and *N. tabacum* cells in suspension cultures. As well as monitoring cell and nuclear area, we have now filled the gap in morphometric analysis showing here that nuclear positioning is affected by the NaCl treatments.

## Materials and Methods

Calli from leaves of *Medicago truncatula* cv. Jemalong line A17 were subcultured monthly on MS medium (Murashige and Skoog, 1962) supplemented with 2.0 mg/l NAA (1-naphthaleneacetic acid), 0.5 mg/l BAP (6-benzylaminopurine) and 3% (w/v) sucrose; pH was adjusted to 5.8 before addition of 0.9% (w/v) agar (MANA medium). Media were autoclaved for 20 min at 121^∘^C/1 par. Cultures were kept at 24/22^∘^C with a 16/8 h (light/dark) photoperiod of 90 μE m^–2^s^–1^ from warm white fluorescent tubes, as reported previously (Elmaghrabi and Ochatt, 2006; Elmaghrabi et al., 2013, 2017).

After 5 months of callus induction on MANA medium, 0.5 g fresh weight pieces of callus were transferred into 250 ml Erlenmeyer flasks containing 100 ml of BY-2 liquid medium and used to establish cell suspensions. BY-2 liquid medium (Nagata et al., 1992) consists of MS (Murashige and Skoog, 1962) medium modified with 0.2 mg/l 2,4D, 1 mg/l Thiamine-HCl, 100 mg/l Myo-inositol and enriched with 200 mg/l KH_2_PO_4_. Cell suspensions were sub cultured every 2 weeks. After four subcultures, once proliferation of suspension cells stabilized, cells were sub cultured into the same medium with a low concentration of NaCl (0, 35, 50, and 70 mM) for gradual acclimation to salt-stress. One month later, these concentrations were changed to 0, 50, 100, and 150 mM NaCl and suspension cultures were maintained as above. Tobacco (*Nicotiana tabacum*) BY-2 cell cultures were analyzed as a comparison to the *M. truncatula* suspension cultures, by adding the same NaCl concentrations to BY-2 liquid medium. Cell suspension cultures of both species were shaken (130 rpm) and were sub cultured every 14 days (Elmaghrabi and Ochatt, 2006).

The viability of the cell suspension cultures was tested by dual propidium iodide (PI) and flouroscein diacetate (FDA) viability staining. The dual stain contained PI (0.24 mg ml^–1^) and FDA (0.04%) and sucrose (w/v, 2%). The cell suspension (75 μl) was added to 75 μl of dual staining solution and incubated on ice for 20 min. Percentage cell mortality was counted using an Olympus BH2 fluorescent microscope at 20× magnification. Approximately 300 cells were scored as either living (green) or dead (red). Culture viability was also assessed by measuring cell density using a spectrophotometer at 600 nm and visually by increasing density of the culture during the 2 week subculture period.

Hoechst staining (1 μL of a 10 mg ml^–1^ stock of Bisbenzimide H, 2 μL Triton X-100 and 97 μL sterile distilled H_2_O) was used to assess cell morphology, using an Olympus BH-2 compound microscope equipped with UV epi-fluorescence. Following 60 days of acclimation to increased salinity, cell and nuclear size were measured using Sigmascan-pro (objective: DPlan Apo 20 UV, 0.70, 160/0.17). Position of nuclei within cells was determined using ArchimedPro and Histolab software (Microvision, France) by six measurements 60° apart for each cell, *n* = 13–19 cells (Supplementary Figure 1).

Data were analyzed using R software (R version 3.3.2, Foundation for Statistical Computing). ANOVA tests followed by a Tukey’s test, or non-parametric Kruskal–Wallis followed by a Dunn’s test were applied to determine differences across multiple samples.

## Results

**FIGURE 1:**
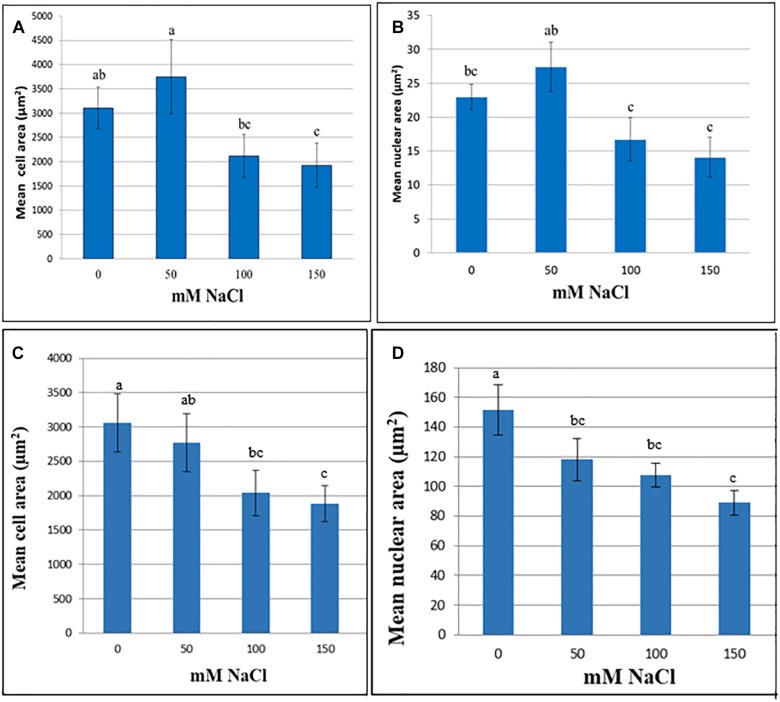
Cell **(A,C)** and nuclear **(B,D)** area (μm^2^) at various NaCl concentrations of cell suspensions of *M. truncatula* **(A,B)** and *N. tabacum* **(C,D)** measured at 60 and 8 days, respectively, following the start of treatment (mean ± SE, *n* = 12). Letters above bars denote significant differences in area between NaCl concentration datasets (*P* < 0.05).

In *M. truncatula* cell suspensions, the initial trend was an increase in cell and nuclear area following 60 days exposure to 50 mM NaCl although these increases were not significant (*P* > 0.05). Likewise, in the tobacco cultures cell area remained stable up to 50 mM NaCl, although nuclear area was already significantly lower than the control (Figure 1). When the NaCl concentration was raised further, to 100 or 150 mM, both nuclear and cell area decreased in both species, perhaps as a function of plasmolysis. Cell area was significantly lower than the control at 150 mM in both species, and nuclear area was significantly lower than the control in both the 100 and 150 mM NaCl treatments (*P* < 0.05; Figure 1). This decline in cell and nuclear size across treatments, despite the presence of viable cells even at the highest NaCl concentration (Supplementary Table 1), and the lack of response at 50 mM NaCl suggests that these traits are not a reliable criterion to assess cell growth of *M. truncatula* or tobacco in response to NaCl stress over a longer period. Similar trends were noted for both cell and nuclear area when suspensions were cultured under the same conditions for up to 4 months (Supplementary Figures 2, 3). Moreover, they showed the opposite trend to those observed for osmotic stress-resistant cells of *M. truncatula* where osmotic stress provoked an increase in cell and nuclear area concomitant with endoreduplication (Elmaghrabi et al., 2017).

**FIGURE 2:**
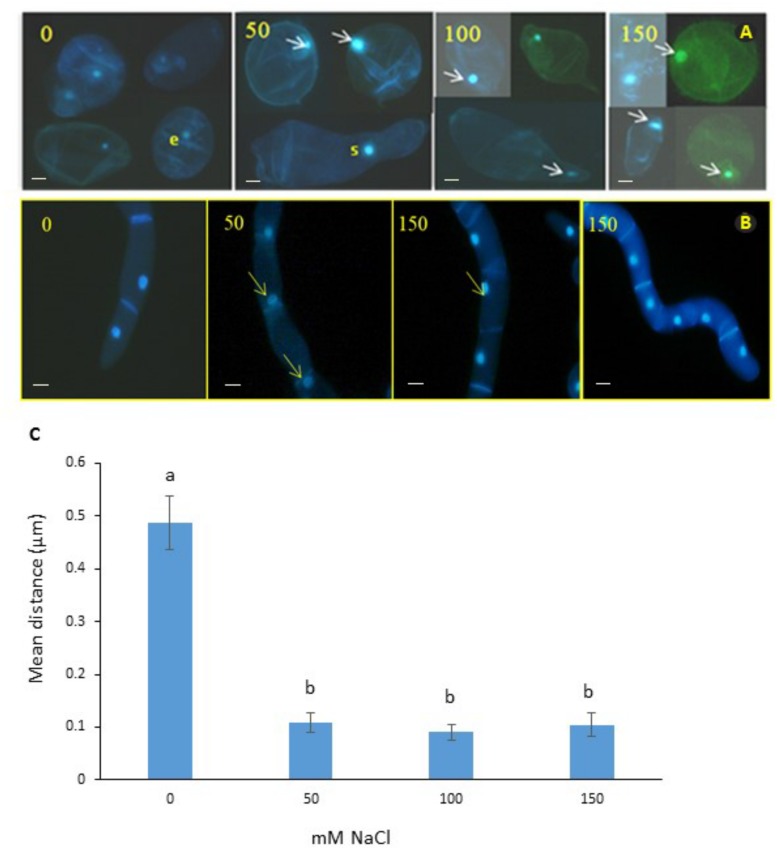
Nuclear positioning in cell suspensions of *M. truncatula* **(A)** and *N. tabacum* **(B)** at different concentrations of salt (mM NaCl). Arrows show migration of the nuclei to the cell wall and elongation of cytoplasm in the 50 and 100 mM NaCl treatments. It is unlikely that this cellular phenotype has embryogenic potential; e, denotes a small cell with a small nucleus in the 0 mM NaCl treatment that might have embryogenic potential; and s, indicates septum forming along the presumptive cell plate of a cell undergoing cytokinesis. Bar scale = 20 μm. **(C)** Position of nucleus in *M. truncatula* cells exposed to NaCl (0–150 mM). Means of six measurements from nuclear envelope to cell wall for each cell + SE; *n* = 13–19 cells (see Supplementary Figure 1). Different letters indicate significantly different means (Kruskal Wallis followed by a Dunn’s test, *P* < 0.05).

We observed mitoses in the *M. truncatula* cell suspensions established from callus for monitoring cellular behavior under salt stress (0, 50, 100, or 150 mM NaCl; Figure 2A). Our aim was to identify a characteristic cellular/nuclear phenotype as a consistent marker of fast growing or salt tolerant callus, for use as a diagnostic criterion of acquisition of *in vitro* salt tolerance in *M. truncatula*, as recently observed under osmotic stress (Elmaghrabi et al., 2017).

Interestingly, subjecting the cell suspension cultures to the 50, 100, and 150 mM NaCl treatments for 2 months consistently resulted in the migration of nuclei from the center toward the periphery of cells (Figure 2C and Supplementary Table 2). To test whether this was a distinctive feature of *M. truncatula* or a more general response to salt stress by plant cells, the tobacco cells were also analyzed, and the same effect was seen (Figure 2B). This repositioning was never observed for the control cells of either species studied when grown under stress-free conditions, where the nucleus maintained a central position within the cytoplasm, equidistant to the wall (Figures 2A,B, first panel). It is also noteworthy in this respect, that these observations were undertaken on cell suspensions that had undergone already the repeated cycles with and without NaCl during the step-up protocol through which they were produced, which would suggest that the phenomenon of nucleus repositioning is correlated to the acquisition of salt tolerance.

## Discussion

In addition to being a model species, *M. truncatula* (barrel medic) can fix atmospheric nitrogen, has high protein content (Young and Udvardi, 2009) and includes cultivars with relatively high salinity tolerance (Merchan et al., 2003). We developed a method for induction of new accessions of *M. truncatula* tolerant to salinity induced by NaCl (Elmaghrabi et al., 2013) and also to osmotic stress provoked by PEG 6000 (Elmaghrabi et al., 2017), in both cases through *in vitro* selection via a step-up recurrent strategy. A gradual exposure to successively higher NaCl concentrations has led to long-term acclimation of cells to salinity in other plant species, and embryogenic and organogenic callus produced by this method have enabled selection of salt resistant cultures (Miki et al., 2001; Merchan et al., 2003). Long-term culture under salt stress conditions may simultaneously induce physiological adaptation in cells (Naliwajski and Skłodowska, 2014) and the generation of acclimated and truly tolerant somaclones (Arzani, 2008), which are then capable of growth at NaCl concentrations that are lethal to non-acclimated ones. In this respect, the continuous assessment of cell viability with time in culture and following our step-up recurrent strategy resulted in a gradual enrichment in truly tolerant cells in the population selected under NaCl stress and the concomitant death of those cells that were only physiologically adapted to the stress imposed (Elmaghrabi et al., 2013).

Here using cell suspension cultures, we explored how exposure to increasing salinity over long culture periods affected cell morphology, and demonstrate that an alteration of nuclear position was more sensitive to low NaCl concentrations than changes in nuclear or cell area. Here we only assessed a single variety of *M. truncatula*, and it will be interesting for future studies to assess different genotypes and species of *Medicago* known for their differential responses to salinity stress, as shown with *M. sativa* (Ehsanpour and Fatahian, 2003; Quan et al., 2016). However, nuclear repositioning in response to NaCl was shown here to be consistent across two very different species, *M. truncatula* and *N. tabacum* suggesting that it may be a widespread plant cellular response.

Understanding of the cellular significance of nuclear position within cells in terms of both their metabolism and physiology is still in its infancy, and all studies on the movement of cells and positioning of their nuclei thus far have been restricted to human and animal cells (Gundersen and Worman, 2013). Among them, in cancerous cells, nuclear positioning was shown to alter their ability to respond to the pathways regulating transcription and mRNA transport and localization (Calvo et al., 2010). It was also speculated that the distance the nuclei traveled depended on various cytoplasmic stimulatory and inhibitory factors, whereby their change of position relative to the origin of an external signal may modulate the nuclear response particularly when signaling is asymmetrical. However, only one study in zebrafish has examined the relationship between nuclear position and asymmetrical signaling (Del Bene et al., 2008). Our results with salt-tolerant plant cells are in line with the studies above. In zebrafish gradients of external stress-inducing factors during development, resulted in a repositioning of the nucleus within the cytoplasm so that its responsiveness to stress might be improved by replacing the nucleus in the close proximity to the stress signal.

Such migration of nuclei from the center of cells toward the outside is a type of perturbation not shown before in plant cells to our knowledge, and appears to be a major effect of salt at the cellular level perhaps related to negative growth responses to the increasing internal concentrations of NaCl of cells in culture. Nuclear migration will be concomitant with the typical cell responses to osmotic stress, including changes in cell wall thickness, vacuole volume and plastid rearrangements as observed in Arabidopsis (Gobert et al., 2007), but also in the surface area of cells and their nuclei as recently reported in osmotic stress resistant cells of *M. truncatula* (Elmaghrabi et al., 2017).

Exposure to high salt stress can induce rapid nuclear deformation (Katsuhara and Kawasaki, 1996) leading to programmed cell death. Moreover, growth of barley at 192 mM NaCl resulted in chromatin condensation (Werker et al., 1983). Nuclear marginalization has also been associated with cell death (O’Brien et al., 1998), suggesting that some of the cells under salt stress in this study are preparing to undergo cell death. On the other hand, *M. truncatula* cell suspensions were shown to respond to stress like whole plants (Elmaghrabi et al., 2013; Araújo et al., 2016) and, in this context, nuclear marginalization as observed would be a part of an eustress cellular mechanism to cope with the induced stress. In this respect, eustress is an activating, stimulating stress, which is a positive element in plant development, and is also referred to as good stress or constructive stress that can promote plant defense secondary metabolisms for improving tolerance to further stress (Kranner et al., 2010; Hideg et al., 2013).

## Conclusion

In conclusion, cell and nuclear size decreased at high NaCl, consistent with signs of plasmolysis, but were not useful traits in explaining cell survival and growth at high NaCl concentrations. Conversely, nuclear marginalization was for the first time observed as a result of salt stress in plant cells, and could be a novel and helpful morphological indicator for acquisition of salinity tolerance. Importantly, our results strongly suggest that the repositioning of the nucleus within the cytoplasm is not passive nor random. Indeed, it results from the onset under stress of a mechanism that may be a common response across eukaryotes.

## Author Contributions

DF, HR, and SO designed the project and experiments. AE performed the experiments. SO and HR wrote and revised the manuscript. All authors analyzed the data, read, and approved the manuscript.

## Conflict of Interest Statement

The authors declare that the research was conducted in the absence of any commercial or financial relationships that could be construed as a potential conflict of interest.
