# Supplementary material for: Nuclear Migration: An Indicator of Plant Salinity Tolerance in vitro
Source: Front Plant Sci. 2019 Jun 12;10:783. doi: 10.3389/fpls.2019.00783 (PMC6582401; doi:10.3389/fpls.2019.00783)
Supplement: Supplementary file 1 [file Presentation_1.PPTX]

## Slide 1
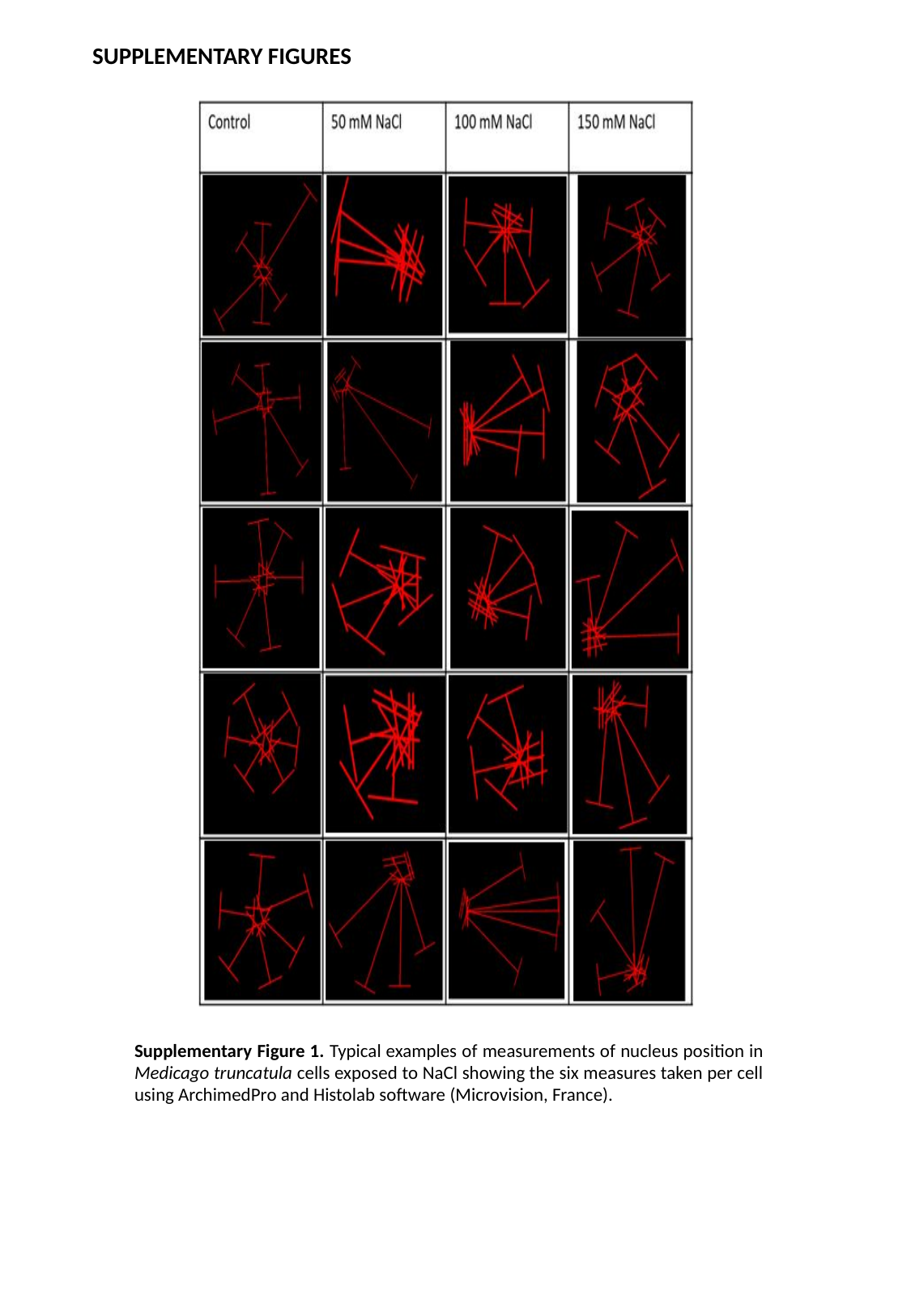

SUPPLEMENTARY FIGURES
Supplementary Figure 1. Typical examples of measurements of nucleus position in Medicago truncatula cells exposed to NaCl showing the six measures taken per cell using ArchimedPro and Histolab software (Microvision, France).

## Slide 2
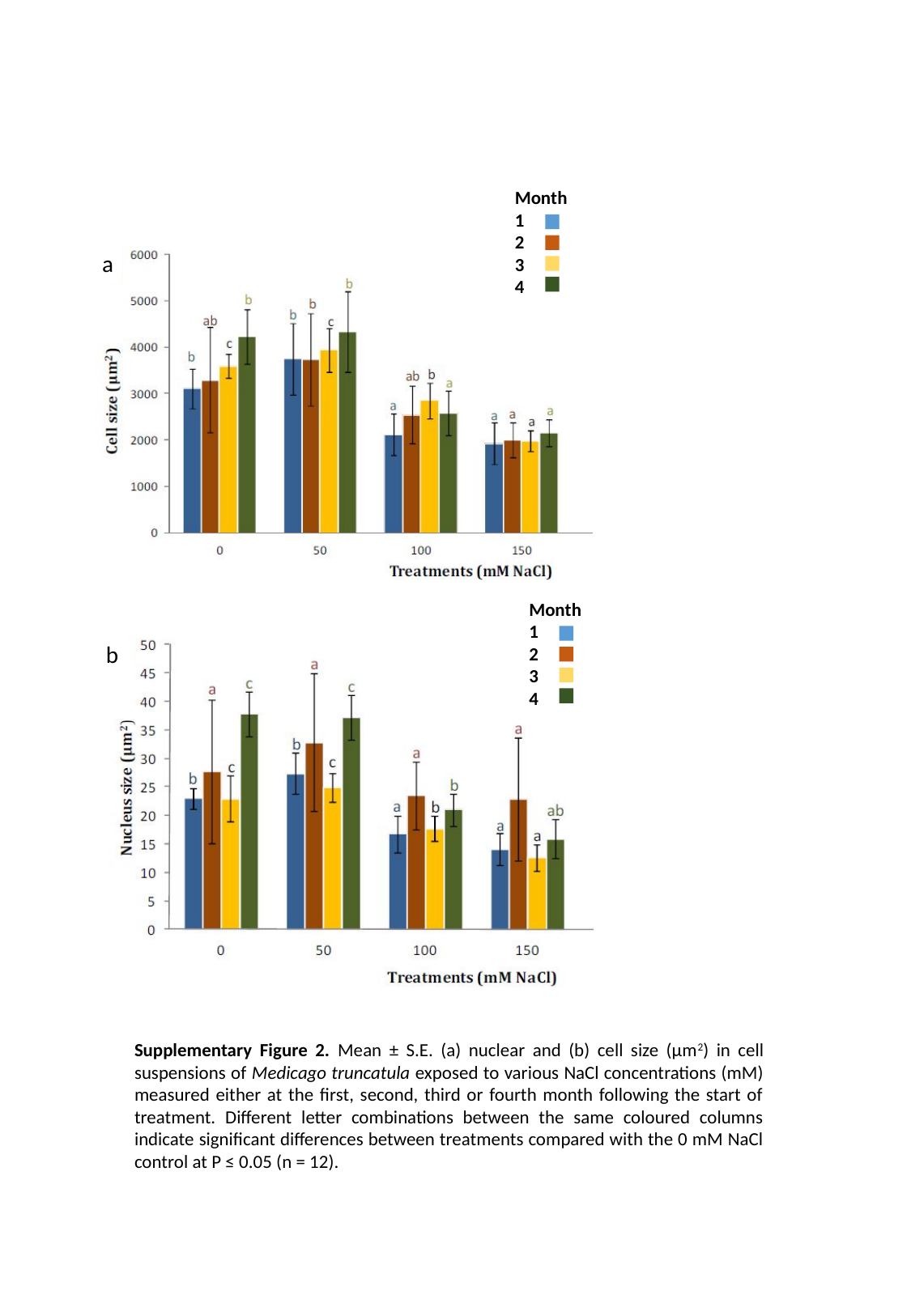

Month
1
2
3
4
a
Month
1
2
3
4
b
Supplementary Figure 2. Mean ± S.E. (a) nuclear and (b) cell size (μm2) in cell suspensions of Medicago truncatula exposed to various NaCl concentrations (mM) measured either at the first, second, third or fourth month following the start of treatment. Different letter combinations between the same coloured columns indicate significant differences between treatments compared with the 0 mM NaCl control at P ≤ 0.05 (n = 12).

## Slide 3
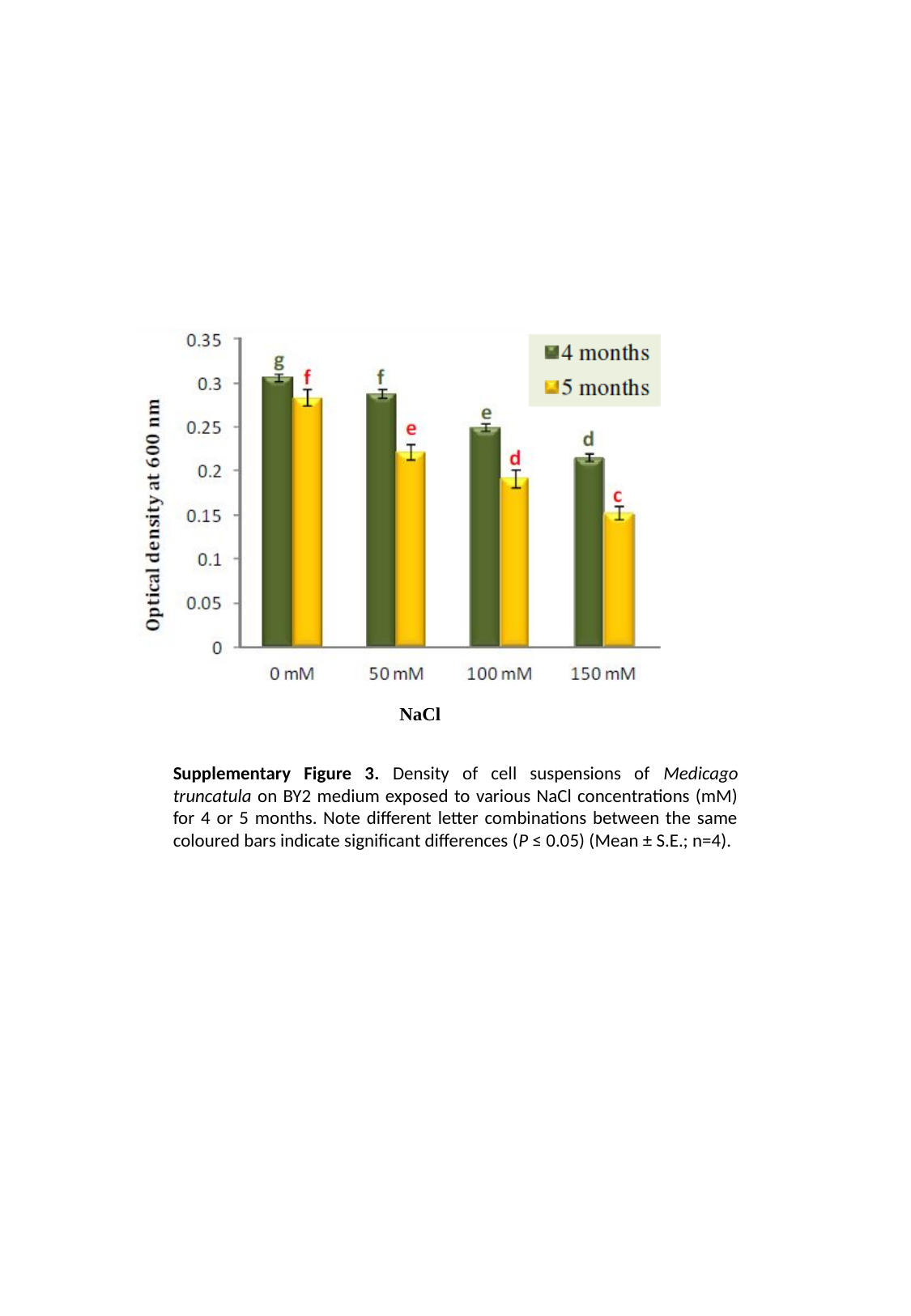

NaCl
Supplementary Figure 3. Density of cell suspensions of Medicago truncatula on BY2 medium exposed to various NaCl concentrations (mM) for 4 or 5 months. Note different letter combinations between the same coloured bars indicate significant differences (P ≤ 0.05) (Mean ± S.E.; n=4).

## Slide 4
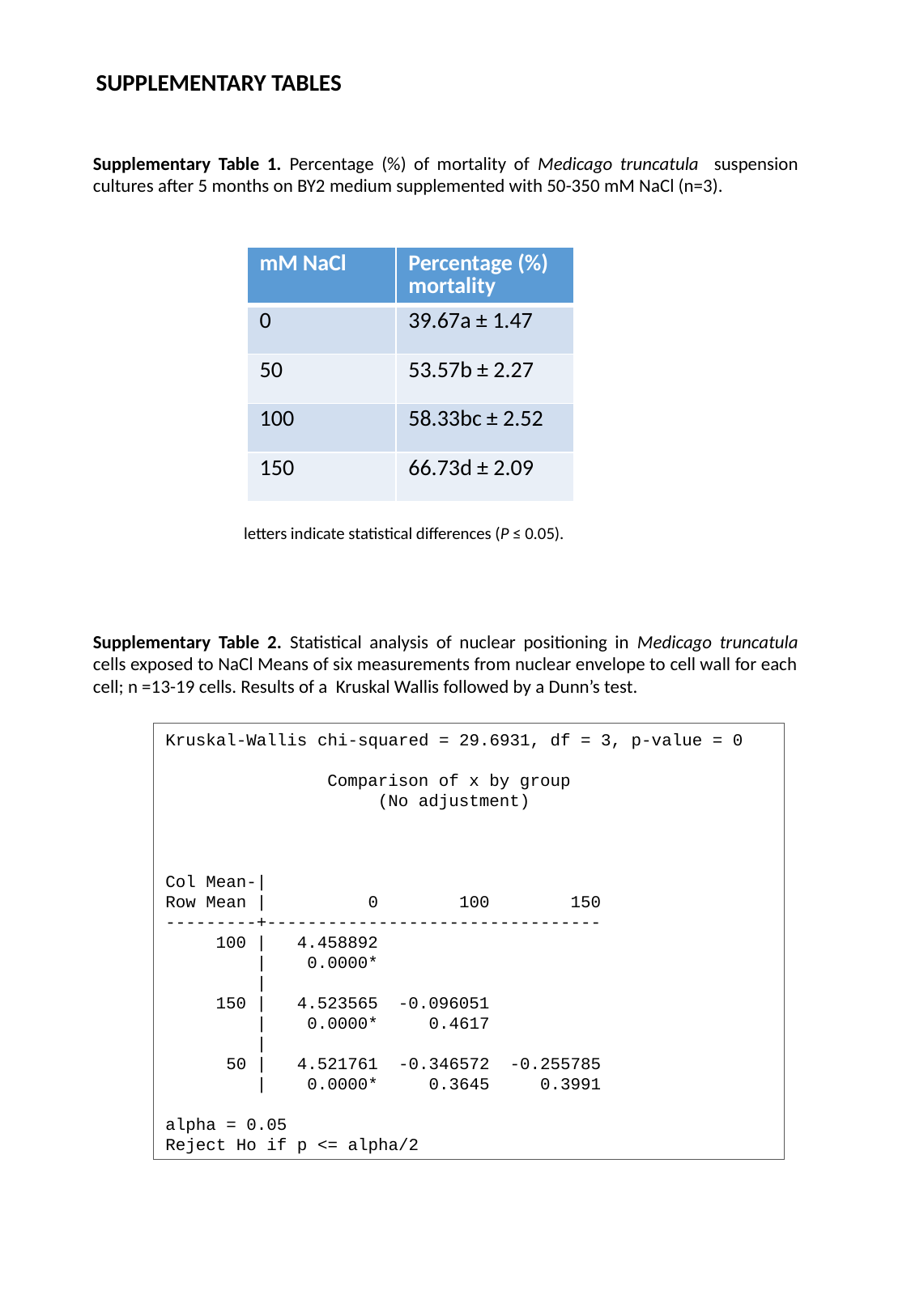

SUPPLEMENTARY TABLES
Supplementary Table 1. Percentage (%) of mortality of Medicago truncatula suspension cultures after 5 months on BY2 medium supplemented with 50-350 mM NaCl (n=3).
| mM NaCl | Percentage (%) mortality |
| --- | --- |
| 0 | 39.67a ± 1.47 |
| 50 | 53.57b ± 2.27 |
| 100 | 58.33bc ± 2.52 |
| 150 | 66.73d ± 2.09 |
letters indicate statistical differences (P ≤ 0.05).
Supplementary Table 2. Statistical analysis of nuclear positioning in Medicago truncatula cells exposed to NaCl Means of six measurements from nuclear envelope to cell wall for each cell; n =13-19 cells. Results of a Kruskal Wallis followed by a Dunn’s test.
Kruskal-Wallis chi-squared = 29.6931, df = 3, p-value = 0
 Comparison of x by group
 (No adjustment)
Col Mean-|
Row Mean | 0 100 150
---------+---------------------------------
 100 | 4.458892
 | 0.0000*
 |
 150 | 4.523565 -0.096051
 | 0.0000* 0.4617
 |
 50 | 4.521761 -0.346572 -0.255785
 | 0.0000* 0.3645 0.3991
alpha = 0.05
Reject Ho if p <= alpha/2
